# Supplementary material for: Silencing of transposable elements may not be a major driver of regulatory evolution in primate iPSCs
Source: eLife. 2018 Apr 12;7:e33084. doi: 10.7554/eLife.33084 (PMC5943035; doi:10.7554/eLife.33084)
Supplement: Supplementary file 1. [file elife-33084-supp1.docx]

**Supplemental File 1**

**Silencing of transposable elements may not be a major driver of regulatory evolution in primate induced pluripotent stem cells**

Michelle C. Ward^1,2*^, Siming Zhao^1^, Kaixuan Luo^1^, Bryan J. Pavlovic^1^, Mohammad M. Karimi^3,4^, Matthew Stephens^1,5^, Yoav Gilad^1,2*^

TABLE OF CONTENTS

Table 1: Details of experimental samples used.

Table 2: Processing of ChIP-seq and RNA-seq samples.

Table 3: ChIP-seq and RNA-seq sequencing data.

Table 4: Differential enrichment analysis of H3K9me3 regions is consistent across low read count filtering methods

Table 5: Differential enrichment analysis of H3K9me3 regions is consistent across two methods.

Table 6: Species-specific TEs in the human and chimpanzee genomes.

Table 7: Differentially expressed genes with differential H3K9me3 silencing of TEs within 1 kb upstream of the TSS.

Table 8: Gene expression divergence between categories of silenced TEs is consistent across various FDR thresholds.

Table 9: Inter-species divergence in H3K9me3 does not explain inter-species divergence in gene expression and *vice versa*.

Table 10: TE expression and overlap with H3K9me3.

Table 11: Differentially expressed orthologous KRAB-ZNFs.

**Table 1: Details of experimental samples used.**

**Table 2: Processing of ChIP-seq and RNA-seq samples.** Samples were processed so as to not confound species (human:blue and chimpanzee:black) with processing step. **(A)** Sample processing of ChIP-seq samples. Numbers denote batch at each step. **(B)** Sample processing of RNA-seq samples.

**Table 3: ChIP-seq and RNA-seq sequencing data.** Summary of sequencing data of human samples (blue) and chimpanzee samples (black). The number of libraries pooled together per sequencing lane is shown in column 4 with the number of lanes sequenced using that pool shown in parentheses. The total number of sequencing lanes is shown in column 5. Read numbers represent the total numbers of paired-end, quality-filtered mapped reads. ‘All peaks’ represent all peaks identified in that individual, and ‘Ortho peaks’ the number of peaks within human-chimpanzee orthologous regions.

**Table 4: Differential enrichment analysis of H3K9me3 regions is consistent across low read count filtering methods.** Number and proportion of H3K9me3 regions identified as being differentially enriched between human and chimpanzee at an FDR of 1% using *DESeq2* and different low count filtering methods. Row 1) Requiring at least half of all individuals to have greater than 0 H3K9me3 read counts in a given region independent of species. Row 2) Requiring at least half of all individuals within a species to have greater than 0 H3K9me3 read counts in a given region. Row 3) Requiring at least half of all individuals to have greater than 2 H3K9me3 read counts in a given region independent of species.

**Table 5: Differential enrichment analysis of H3K9me3 regions is consistent across two methods. (A)** The number and proportion of H3K9me3 regions that are differentially enriched (DE) between human and chimpanzee using either *DESeq2* or *voom+limma* is similar across different FDR thresholds**. (B)** The number and proportion of total H3K9me3 regions differentially enriched at different effect sizes using *DESeq2***.**

**Table 6: Species-specific TEs in the human and chimpanzee genomes.**

**Table 7: Differentially expressed genes with differential H3K9me3 silencing of TEs within 1 kb upstream of the TSS**

**Table 8: Gene expression divergence between categories of silenced TEs is consistent across various FDR thresholds.** FDR thresholds used to classify differentially enriched windows of H3K9me3-mediated TE silencing as Shared (S), Human-enriched (H), and Chimpanzee-enriched (C) considering 10, 20 and 40 kb windows upstream of the TSS. *P* values determined by the Wilcoxon-rank sum test between categories. All *P*-values > 0.01.

**Table 9: Inter-species divergence in H3K9me3 does not explain inter-species divergence in gene expression and *vice versa*. (A)** Proportion of genes that are considered to be differentially expressed (DE) between species at a FDR threshold of <1% when varying the FDR threshold to identify inter-species differences in H3K9me3 upstream of the gene. *P* values from the Pearson’s Chi-square test are shown. The proportion of all genes, irrespective of upstream H3K9me3 enrichment, that are differentially expressed between species at FDR < 1% is shown in the bottom row. **(B)** Reciprocal analysis of (A) where the FDR threshold used to identify differentially expressed genes is varied. The proportion of all H3K9me3 regions, irrespective of whether upstream from a gene, that are differentially expressed between species at FDR < 1% is shown in the bottom row. DE: H3K9me3 windows that are differentially enriched between species, or genes that are differentially expressed. nonDE: windows/genes that are not differentially enriched/expressed between species.

**Table 10: TE expression and overlap with H3K9me3. (A)** TE types that are expressed (RPKM >1) in each species. All instances of each type in each species were collapsed to yield a single RPKM value per type. **(B)** Instances of TE types that are orthologous in each species and have the potential to be expressed in at least one species together with the overlap with H3K9me3 regions.

**Table 11: Differentially expressed orthologous KRAB-ZNFs.** KRAB-ZNFs with an inter-species log Fold Change < -1 or >1, and adjusted *P* value < 0.01 are shown.
